# Cross Talk between Viruses and Insect Cells Cytoskeleton

**DOI:** 10.3390/v13081658

**Published:** 2021-08-20

**Authors:** Ayda Khorramnejad, Hugo D. Perdomo, Umberto Palatini, Mariangela Bonizzoni, Laila Gasmi

**Affiliations:** Department of Biology and Biotechnology, University of Pavia, Via Ferrata 9, 27100 Pavia, Italy; ayda.khorramnejad@unipv.it (A.K.); hugo.perdomo@unipv.it (H.D.P.); umberto.palatini01@universitadipavia.it (U.P.); mariangela.bonizzoni@unipv.it (M.B.)

**Keywords:** cytoskeleton, virus motility, baculovirus, vector–virus interactions

## Abstract

Viruses are excellent manipulators of host cellular machinery, behavior, and life cycle, with the host cell cytoskeleton being a primordial viral target. Viruses infecting insects generally enter host cells through clathrin-mediated endocytosis or membrane fusion mechanisms followed by transport of the viral particles to the corresponding replication sites. After viral replication, the viral progeny egresses toward adjacent cells and reaches the different target tissues. Throughout all these steps, actin and tubulin re-arrangements are driven by viruses. The mechanisms used by viruses to manipulate the insect host cytoskeleton are well documented in the case of alphabaculoviruses infecting Lepidoptera hosts and plant viruses infecting Hemiptera vectors, but they are not well studied in case of other insect–virus systems such as arboviruses–mosquito vectors. Here, we summarize the available knowledge on how viruses manipulate the insect host cell cytoskeleton, and we emphasize the primordial role of cytoskeleton components in insect virus motility and the need to expand the study of this interaction.

## 1. Introduction

Insects are the most diverse and successful animal group on earth and host a huge diversity of viruses ranging from entomopathogenic viruses (EPVs), which cause lethal infections, to insect-specific viruses (ISVs), which cause non-lethal persistent infections. In addition, insects can act as vectors for most plant viruses and a wide range of veterinary and public health-relevant pathogenic viruses. Viruses that establish persistent infections in insect hosts must overcome several barriers before being transmitted to a secondary host. These barriers may include viral replication and/or dissemination in the host gut, trachea, salivary glands, and/or ovaries. In general, viruses enter the host cells by fusion of their viral envelope with the plasma membrane or via endocytosis followed by their subsequent escape from endosomes by membrane fusion or lysis, allowing the delivery of viral particles to the corresponding replication sites [1]. The dynamics of viral dissemination from cells of the initially infected tissue are driven by re-arrangements of tubulin and/or actin filaments that result in the regulation of cytoskeleton dynamics [2].

The cytoskeleton and membrane systems of eukaryotic cells play key roles in the intracellular transport of vesicles, organelles, and macromolecules. These features are targeted by infecting viruses. As early as the 1970s, viral particles were shown to localize within cytoskeleton elements, and disruption of the cytoskeleton negatively affected viral replication [1]. Both the microtubule and actin cytoskeletons participate in active transport at multiple steps of the infection cycle of animal viruses, including the binding of virions to receptors, internalization, movement to the different cell compartments, genome replication, virion assembly, and virus egress [3,4]. At different stages of the viral replication cycle, particle motility inside the cell and between cells is needed. How viruses manipulate the host cell cytoskeleton to move from cell to cell or within the cytoplasm is understood only in few virus–insect host systems, primarily regarding baculoviruses and their Lepidopteran hosts. The same is true for processes allowing viral particles to enter the nucleus and egress toward further target tissues. Herein, we summarize the current knowledge on viral manipulation of the cytoskeleton of insect cells and highlight the need for further studies.

## 2. The Cytoskeleton in the Context of Animal–Virus Interactions

### 2.1. The Actin Cytoskeleton

The actin cytoskeleton is mainly composed of globular actin (G-actin), which is monomeric actin able to self-assemble into filamentous actin (F-actin). The formation of new actin filaments requires different actin regulators, one of which is the actin-related protein 2/3 (Arp2/3) complex [5]. Changes in Arp2/3 affect the overall organization of the cytoskeleton network [5]. The filamentous network formed by F-actin and actin-binding proteins is the primary component of cytoskeleton microfilaments [6]. The actin cytoskeleton is subjected to alterations and organizations to promote cellular dynamic and particle transport within and between cells. Eukaryotic cells polymerize actin filaments to provide mechanical integrity and motility force for a wide range of cellular mechanisms [7].

Most if not all animal viruses studied so far manipulate the cellular actin from the first steps of their infection cycle. The binding of virions to specific receptors and entry into host cells is mediated by the cortical network of actin filaments, which are involved in the internalization of the virions localized at the surface of the host cells [8,9,10]. The dynamic polymerization of actin reshapes cellular membranes allowing viral internalization and has a role in the formation and movement of endocytic vesicles in case of entry through endocytosis mechanisms [11]. Host actin is also manipulated by viruses during the intracellular movement of the viral genome and proteins, and the delivery of the viral particles to the corresponding replication sites [12,13]. Finally, the actin cytoskeleton is required for viral egress and dissemination of the virus to other host tissues [14,15,16].

### 2.2. Microtubule Cytoskeleton

Microtubules are formed through α-/β-tubulin heterodimers assembling into cylindric filaments. The plus-ends of these filaments grow pointing towards the plasma membrane into protrusions, while their minus ends are anchored at microtubule-organizing centers (MTOCs) such as the centrosome [17,18]. This polarity allows selective directional long-range cargo transport at the cell periphery [18].

**Figure 1:**
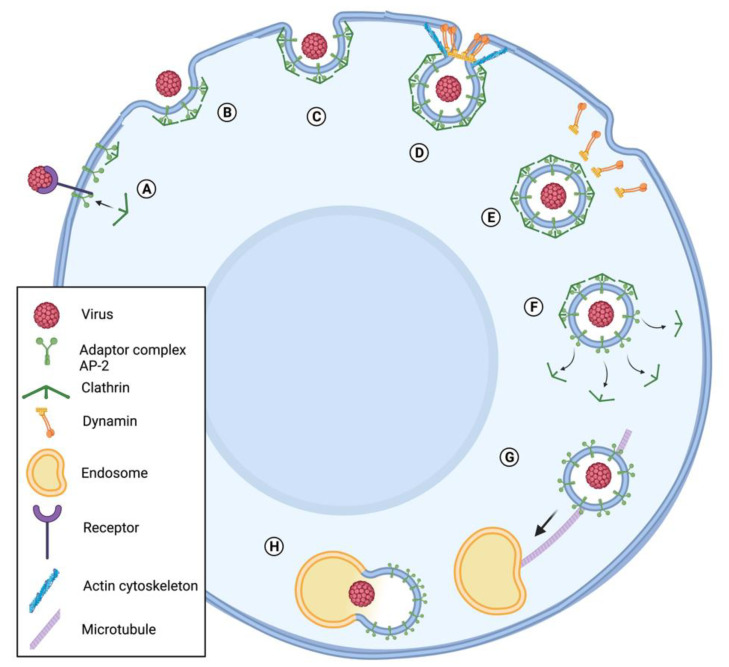
Viral internalization in host cell through clathrin-mediated endocytosis. The virus binds to a receptor, initiating a series of steps that lead to endocytosis. (**A**) The adaptor protein complex AP2 links clathrin to the membrane, recruits other accessory proteins important for the endocytosis, and assembles a clathrin coat. (**B**) The clathrin coat grows, leading to invaginations in the membrane (**C**) and later to the formation of a vesicle. (**D**) The vesicle is pinched off by a mechanism mediated by dynamin and the actin cytoskeleton. (**E**) After the vesicle detaches from the cell membrane, (**F**) it loses the clathrin coat (**G**) and travels to an endosome with the help of microtubules. (**H**) The vesicle binds to an endosome and starts endosome maturation. Adapted from “Clathrin-Mediated Endocytosis”, by BioRender.com (2021). The template of this figure was retrieved from https://app.biorender.com/biorender-templates (Publication License Agreement Number CN22UJ223Y).

The microtubule network is often used by viruses in the first steps of attachment into specific sites of host cell receptors. The spatial reorganization of the receptors and viral entry is in part controlled by this microtubule organization at the cell surface [19,20,21]. One of the most recognized and characterized viral internalization mechanisms is clathrin-mediated endocytosis. Clathrin is a self-assembling protein that coats transport vesicles by polymerizing into polyhedral lattice. These formations are called coating pits and contribute to the organization and sorting of integral membrane proteins during receptor-mediated endocytosis and organelle biogenesis and is involved in several cellular pathways such as cargo transport into the cells, protein secretion from the trans-Golgi network, and signal transduction [22,23,24]. Following viral particles’ attachment to the host cell surface, these are delivered to pre-formed clathrin-coated pits (Figure 1). Then, these pits pinch off from the plasma membrane through a mechanism controlled by cytoskeleton-specific proteins, including actin [24] (Figure 1). Following internalization, clathrin pits gradually disassemble, delivering viral particles to endosomes, which travel across the cytoplasm through microtubule-related mechanisms (Figure 1). The trafficking of coated vesicles to early endosomes, late endosomes, and lysosomes requires microtubules arrangement [25]. Following the endosomes escape, viruses that replicate in the nucleus usually manipulate microtubule dynamics to facilitate the transit into the nucleus [25]. Microtubule-based motor proteins, such as kinesin and dynein, transfer the cargoes along microtubules in cells [26]. Dynein forms a complex with dynactin (actin-related filamentous protein) facilitating the cargo trafficking toward the cell center near the nucleus. Virions’ progeny travel toward microtubule plus ends, from near the nucleus to the peripheral regions of the cell, through kinetin-based microtubule [26,27]. Virions require actin and myosin for short transportation, while microtubules and dynein mediate the long-range transportation [10,26]. RNA viruses use different cytoplasmic replication structures and reorganize microtubules to enhance the formation of replication compartments [17].

## 3. Baculoviruses Hijack the Host Cells Cytoskeleton Actin to Replicate and Spread the Infection

Baculoviruses (*Baculoviridae*, double-stranded (ds)DNA) constitute a unique group of viruses that is specific to arthropods, mainly insects. Baculoviruses are the most studied EPVs that cause epizootic diseases in nature. Baculoviruses are also commercialized as biological pesticides to control major forest and agricultural pests. They are enveloped viruses with a circular, double-stranded DNA genome wrapped in rod-shaped nucleocapsids. Baculoviruses exist in two forms: occlusion-derived viruses (ODVs) and budded viruses (BVs). ODVs contain one or several nucleocapsids and are enveloped in an occlusion body (OB) resistant in the environment. Once the insect ingests a baculovirus-contaminated food and OBs reach the midgut, the alkaline pH dissolves the envelope. Released ODVs bind and fuse to the epithelial cells, releasing the nucleocapsids into the gut cells where they replicate in the nucleus. The viral progeny bud out of the cells, forming BVs that are single enveloped nucleocapsids. BVs spread the infection to adjacent tissues and therefore are responsible for secondary infections. In the final stages of the infection cycle, the viral nucleocapsids progeny gets occluded in a protein matrix to form OBs that are released in the environment upon the death of the insect [28]. The expression of baculovirus proteins is regulated throughout the replication cycle, and three groups of genes can be observed: early genes, which are transcribed by the host RNA polymerase II early on in the infection [29], late genes, and very late genes that are transcribed by the baculovirus RNA polymerase at the late steps of the replication cycle [30]. The transition from early to late gene expression is marked by the beginning of viral replication around 6 h post infection (hpi) [31].

Baculoviruses manipulate host cell properties, taking control of the host cellular machinery throughout their infection cycle. Unlike other insect viruses, the impact of baculovirus infection on the host cell cytoskeleton has been well characterized. Cytoskeleton re-arrangements happen in different steps of the infection and are crucial for baculovirus proper assembly and the encapsulation of newly produced virions [32]. These interactions were mainly characterized for the baculovirus model Autographa californica multiple nucleopolyhedrovirus (AcMNPV) infection in cell lines of the lepidopteran pests *Spodoptera frugiperda* and *Trichoplusia ni*.

### 3.1. Baculovirus Entry and Transport through the Cytoplasm Depend on Re-Arrangement of Host Cell Cytoskeleton

**Figure 2:**
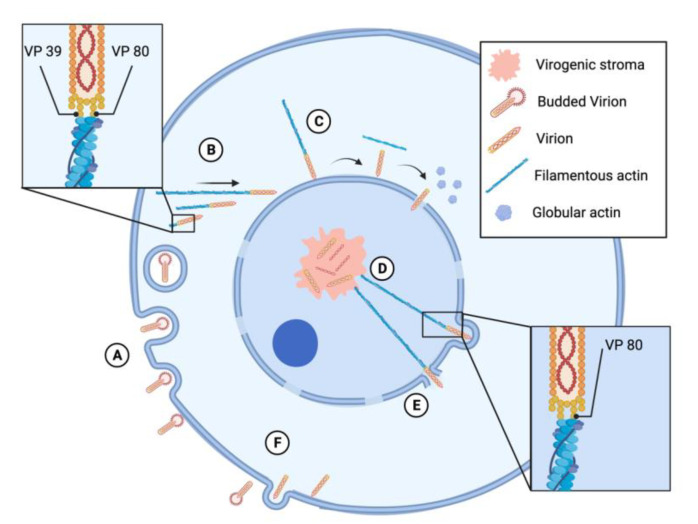
Baculovirus replication cycle and its interaction with actin. (**A**) Budded virions bind to the cell membrane and are endocyted through a clathrin-dependent process. After internalization, the nucleocapsids are released from the endosomes into the cytosol. (**B**) The nucleocapsid proteins VP39 and VP80 bind directly to actin and promote actin polymerization. The viruses are moved to the nucleus via an actin assembly-dependent mechanism, where they collide and stick to the nuclear envelope. (**C**) After the collision, the virions detach from the actin filaments, the filaments depolymerize into globular actin, and the virions enter the nucleus through nuclear pores. Inside the nucleus, the virions move to the virogenic stroma. (**D**) After progeny virions are formed, they are transported to the nuclear periphery by filamentous actin as a result of actin binding to VP80. (**E**) During the egress, baculovirus virions form protrusions in the nuclear envelope and disrupt it. (**F**) Finally, the virions are transported through the cytoplasm and become budded virions through budding of the cytoplasmic membrane. The figure was designed using BioRender (Publication License Agreement Number BR22U9HSSA).

Baculovirus nucleocapsids enter their host cells through clathrin-dependent endocytosis (Figure 1), although in some cases, direct fusion of virions with the host plasma membrane has been described [33,34]. The first cytoskeleton arrangement induced by baculovirus infection happens as soon as the viral particles enter the host cell. Upon internalization of AcMNPV, viral nucleocapsids are released from the endosomes into the cytoplasm (Figure 2). A few minutes post infection, filamentous actin cables form as a result of the interaction between viral nucleocapsid proteins and host actin. The nucleocapsid proteins VP39 and VP80, which have been identified in several baculoviruses such as AcMNPV, *Helicoverpa armigera* NPV (HaNPV), and *Bombyx mori* NPV (BmNPV), bind directly to actin and promote F-actin polymerization [13,35,36] (Figure 2). The sequence of VP39 from 55 currently available baculovirus genomes was analyzed, leading to the identification of a conserved glycine at position 276. Mutation of this Gly^276^ resulted in alterations of correct nucleocapsid assembly, DNA packaging, and expression of late genes [37]. These results altogether suggest that Gly^276^ is primordial for VP39 function and may be indispensable for VP39 interaction with actin and therefore nucleocapsid movement toward the replication sites of the virus. In AcMNPV-infected High Five cells (obtained from ovaries of *T. ni*), viral nucleocapsids moved within the cytoplasm and were followed by actin comet tails at very early time of infection (5–30 min post infection) (Figure 2). This movement was actin-assembly dependent, since it was inhibited by an inhibitor of actin polymerization. The nucleocapsid–actin interaction is promoted by the interaction of the nucleocapsid promotor factor P78/83 and the Arp2/3 complex, as the inhibition of Arp2/3 decreased the velocity of the viral movements and blocked the nuclear import of nucleocapsids [13,38]. Therefore, P78/83 acts as a nucleation promotor factor (NPF) inducing actin polymerization. The C-terminal part of P78/83 is conserved with other NPFs; meanwhile, the N-terminal part contains a multifunctional regulatory sequence (MRS) and is unique to the baculovirus P78/83 protein family. P78/83 stability is modulated by the MRS that prevents the host cell proteolytic machinery from interacting with and degrading P78/83 [12].

Immediately following early gene expression, actin is re-arranged, resulting in actin filaments aggregating at the plasma membrane. Actin cables localize at the cell surface and transport the released nucleocapsids through the cytoplasm toward the nucleus. This arrangement is a result of the interaction of viral actin re-arrangement-inducing factor 1 (arif-1) with F-actin aggregates [39]. Arif-1 is expressed at the early stage of baculovirus infection and co-localizes with F-actin at the plasma membrane until late gene expression. It has been suggested that ARIF-1 induces the formation of invadosome-like structures, leading to the formation of organized structures to enable systemic viral spread [40]. The fast and early translocation of the nucleocapsids to the nucleus is critical for baculovirus replication.

### 3.2. Baculovirus Entry into the Nucleus and Viral Progeny Egression Are Mediated by Actin Re-Arrangements

Baculoviruses rapidly migrate to the nucleus within 1 hpi. Nucleocapsids collide and are stuck with the nuclear envelope (Figure 2). AcMNPV nucleocapsids in Hi5 cells remained docked at the nuclear periphery for 31 min after collision; then, they separated from actin and entered the nucleus through the nuclear pores [13]. Entry of nucleocapsids into the nucleus ended the actin re-arrangements on the nuclear periphery (Figure 2). When nucleocapsids cross the nuclear membrane, P78/83 MRS most probably gets exposed to the cellular proteolytic machinery, allowing the degradation of P78/83. Consequently, the induction of F-actin polymerization at the nuclear periphery gets blocked, and those filaments depolymerize [12]. Following entry into the nucleus, viral particles move to the sites of uncoating and gene expression [13]. In the AcMNPV-infected *S. frugiperda* cell line SF21, nuclear actin-based motility inside the nucleus had started by 12 ± 2 hpi and had been enhanced by 16 ± 3 hpi. This period coincides with the start of development of viral replication centers, which are called virogenic stroma [14].

Globular actin starts to accumulate in the nucleus since early viral gene expression, while F-actin starts to polymerase with the expression of late viral genes. AcMNPV nucleocapsid protein VP80 in infected cells associates with the nuclear induced F-actin, forming a three-dimensional network that connects the replication factories with the viral nucleocapsids [35]. At 12 hpi, VP80 spreads through the nucleus and then starts to co-localize with DNA-containing areas in the virogenic stroma connecting the central area of the nucleus and the nuclear periphery by 24 hpi. These observations suggest that VP80 is primordial for progeny virions egress from the nucleus [35] (Figure 2). The sequences of both AcMNPV and BmNPV VP80 proteins were characterized and compared to those of available protein sequences in several databases [35]. A conserved domain encompassing GLy^164^–Glu^368^ was identified as homologous to the insect paramyosin sequence Glu^168^–Glu^380^, which was identified in *B. mori* and *Aedes aegypti*. In addition, a second conserved motif encompassing Tyr^575^–Phe^597^ was identified and aligned with the calmodulin-binding domain of eukaryotic myosin-motor proteins, which was identified in several invertebrate and vertebrate organisms such as *Drosophila melanogaster* and *Arabidopsis thalina* [35]. Paramyosin proteins form filaments with myosin motor proteins and interact with actin-based filaments to ensure filament-based motility. The presence in VP80 of a domain shared with paramyosin suggests that VP80 is a paramyosin-like protein that mimics the host myosin-promotor proteins, and it interacts with actomyosin filaments to facilitate active and directed nucleocapsid transport [35].

Following progeny nucleocapsids assembly, the nucleocapsids are transported to the nuclear periphery becoming BVs, which bud through the cytoplasmic membrane (Figure 2). The nucleocapsids, which are retained in the nucleus at very late time post infection, get enveloped, are occluded into the envelope protein polyhedra, and form ODVs [41]. Another event of actin re-arrangement favors AcMNPV virions exit from the nucleus. Actin-driven viral motility is necessary to enter protrusions of the nuclear envelope as well as disruption of nuclear envelope integrity during egress [14]. This actin-dependent disruption of the nuclear envelope during the egression of viral progeny is unique to baculovirus.

### 3.3. Baculovirus Interactions with Microtubules

While AcMNPV harnesses actin arrangements throughout all steps of the replication cycle, its effect on microtubules have not been completely unraveled yet. AcMNPV infects host cells via clathrin-mediated endocytosis, releasing nucleocapsids from early endosomes in a microtubule-dependent manner [9].

Additionally, baculoviruses express a nucleocapsid protein, EXON0, that co-localizes with nuclear microtubules, suggesting a possible interaction between microtubules and nucleocapsids at late time post infection. Treating baculovirus-infected *S. frugiperda* Sf9 cells with microtubule inhibitors caused an 85% reduction in BV yield, suggesting that microtubules are essential for BV egression from the nucleus [42]. BV yield reduction is time-dependent and relies on the nucleocapsid form during trafficking [43]. P10 is another baculovirus protein that was suggested to interact with host cell microtubules. In AcMNPV-infected Sf9 cells, P10 formed fibrous-like structures that extend from the cytoplasm to the nucleus as a result of interaction with cellular microtubules. P10 fibrous-like structures result from the aggregation of P10 monomers that associates with the microtubules subunit α-tubilin, forming stronger microtubule bonds [44]. At a late infection time, these structures fragmented in association with plasma membrane disruption and cellular lysis [45,46].

## 4. Examples of Viruses Other Than Baculoviruses That Manipulate Host Cytoskeleton for Successful Transmission

### 4.1. Plant Viruses Transmitted by Insect Vectors

Most plant pathogenic viruses rely on vectors, including insects, for survival and transmission [47]. Viral acquisition, retention, and inoculation periods greatly vary, influencing virus–host interactions [48,49]. Some plant viruses, such as the Cucumber mosaic virus (*Bromoviridae*, positive-sense single-stranded (ss)RNA) and the Lettuce infectious yellows virus (*Closteroviridae*, positive-sense ssRNA) vectored by the aphid *Myzus persicae* and the whitefly *Bemicia tabaci*, respectively, are only retained by the insect vector on the surface of their feeding apparatus and are transmitted to the next plant [50,51]. Meanwhile, other viruses, such as the Rice dwarf virus (RDV, *Reoviridae*, dsRNA) and the Rice black-streaked dwarf virus (*Reoviridae*) transmitted by the leafhopper *Nephotettix cincticeps* and the planthopper *Sogatella furcifera,* respectively, replicate and disseminate in the host establishing a persistent infection before being transmitted to a new host [52,53]. Persistent plant viruses have complex relationships with their host: they circulate sequentially through the insect stylet, esophagus, digestive tract, and gut epithelial cells to reach the hemolymph and circulate toward the salivary glands and ovaries [49]. Throughout this process, plant viruses exploit the host cellular machinery to propagate, move, and complete their life cycle. Breaching through the different physical barriers of the insect vector (i.e., gut, ovaries, salivary glands) is shown to be dependent on re-arrangements of the host cytoskeleton [54,55]. Re-arrangement of the host cytoskeleton has been mostly studied for reoviruses, which are non-enveloped segmented double-strand RNA viruses that are vectored by leafhoppers or planthoppers (Hemiptera: Auchenorrhyncha) [56].

#### 4.1.1. Plant Virus Entry, Transmission, and Breaching through the Host Intestinal Barrier Is Mediated by Viral Interaction with the Host Cytoskeleton

**Figure 3:**
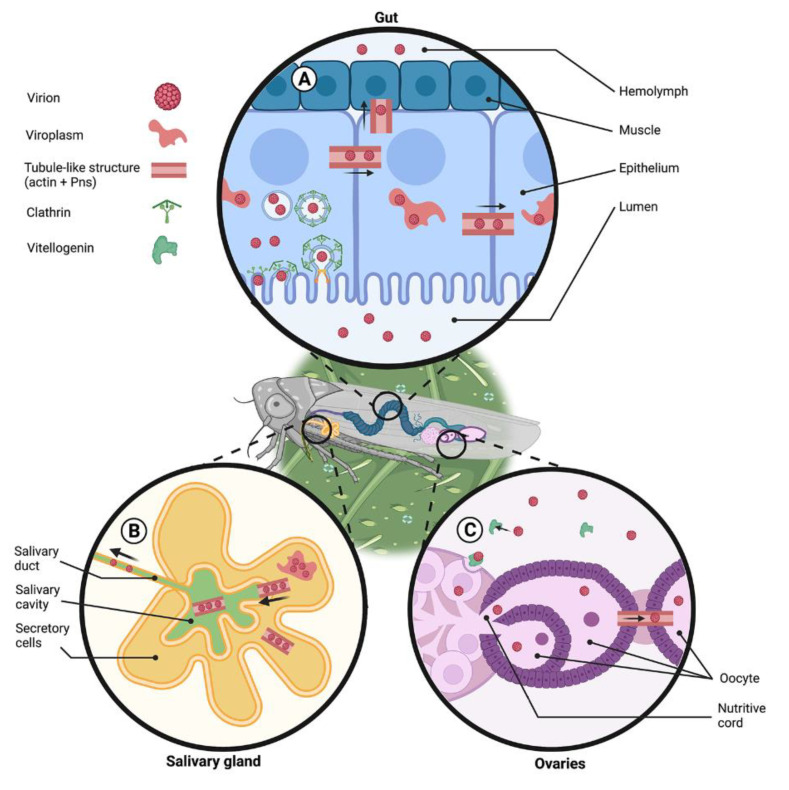
Reovirus infection cycle in the insect vector. Virions should breach three major tissue barriers: insect gut for their dissemination throughout the insect body (**A**), salivary glands for their horizontal transmission (**B**), and ovaries for their vertical transmission (**C**). (**A**) Gut. The virus enters the gut epithelial cells via clathrin-mediated endocytosis. Inside the cell, the virus forms viroplasms, which are subcellular structures where viral accumulation, the synthesis of viral proteins, and replication take place. One of the viral non-structural proteins (Pns) interacts with the insect actin and forms tubule-like structures, which facilitate the infection of neighboring cells and eventual viral release to the hemolymph and viral dissemination. (**B**) Salivary glands. Tubule-like structures are formed by the interaction of actin and Pns in infected secretory cells. These structures are used by the virus to egress from the secretory cells and reach the salivary cavities. Virions present in the cavities are excreted when the insect feeds. (**C**) Ovaries. Virions bind to vitellogenin in the hemolymph and enter the ovaries through vitellogenin-receptor mediated endocytosis. The virus can spread to the cytosol of the oocytes though the nutritive cord or through the tubule like structures, which are formed by interactions of viral proteins and actin that cross the junctions between follicular cells. The figure was designed using BioRender (Publication License Agreement Number TH22QJKFSB).

RDVs penetrate the vector *N. cincticeps* cells via clathrin-mediated endocytosis [57] (Figure 3). The early endosomes directly move to the plasma membrane through the tubular network and release viral particles into the cytoplasm [57,58]. Microscopical observations of RDV motility after internalization showed that the non-structural viral protein Pns10 forms tubule structures through interaction with myosin-motor proteins [59]. The Pns10 tubules directly interact with actin and actin-binding proteins such as myosin, tropomodulin, and vitellogenin [15]. As a result, actin filaments are recruited to enclose tubules of approximately 85 nm in diameter. These tubules protrude from the surface of leafhopper cells and are surrounded by an extended plasma membrane. The virion containing tubules pass through the microvilli of the intestine into the lumen where they associated with actin-based filopodia, and this facilitates viral spread to adjacent tissues [59,60] (Figure 3). Moreover, the inhibition of actin filaments abrogated the extension of Pns10 tubules from the surface of RDV in infected *N. cincticeps* cells. This negatively affected the intercellular spread of RDV, confirming the importance of actin protrusions in the efficient spread of Pns10 tubules from the surface of infected cells to non-infected neighboring cells [60]. The interaction between Pns10 and actin is specific to the natural vector *N. cincticeps* and involves a putative α-helical transmembrane domain encompassing amino acids 98 to 119 of Pns10. If this domain is deleted, it impairs the protrusion of Pns10 tubules from infected cells, thus preventing penetration to neighboring cells [59,60]. There was no interaction between Pns10 and actin of *Recilia dorsalis*, a leafhopper from the same family that is inefficient in transmitting RDV [61]. In a natural host, transmission of RDV from the microvilli of epithelial cells to the visceral muscle tissues of the digestive canal is a result of RDV manipulation of actin-based cellular protrusions (Figure 3). The actin-associated protein tropomodulin positively controls the transmission of RDV tubules by regulating the length and stability of the actin-tropomyosin filament [15]. These data altogether suggest actin-based cellular machinery and actin-associated proteins enable RDV efficient spread from cell to cell and from the gut to adjacent tissues.

Similar viral spread mechanisms have been described for other plant viruses. The rice gall dwarf virus (RGDV: *Reoviridae*), which is transmitted by the leafhopper *R. dorsalis,* forms viral tubules from the non-structural protein Pns11. In host cells, persistent cell-to-cell spread of RGDV occurs through the trafficking of viral tubules along actin-based cellular protrusions of infected cells to neighboring uninfected cells [62]. The southern rice black-streaked dwarf virus (SRBSDV: *Reoviridae*), which is transmitted by the white-backed planthopper *S. furcifera,* also encodes a non-structural protein, P7-1, which arranges virus-containing tubules. The self-interaction of P7-1 monomers leads to the formation of homodimers or oligomers [63], resulting in the formation of the helical structure of viral tubules. These tubules travel along the basal lamina of the intestinal epithelium and the intestine circular muscle fibers by interacting with the actin-based cellular protrusions. As a result, SRBSDV disseminates across the epithelium toward the gut visceral muscle [64]. The beet western yellow virus (BWYV: *Solemoviridae*, positive-sense ssRNA), which is transmitted by aphids, can bind to different host proteins, including actin. BWYV crosses the aphid intestine, hemocoel, and the salivary glands through transcytosis mediated by interaction of the virus with cytoskeleton elements [16].

A different mechanism of interaction with the host cytoskeleton was described for the rice stripe virus (RSV: *Phenuiviridae*, negative-sense ssRNA), which is transmitted by the small brown planthopper *Laodelphax striatellus*. RSV forms filamentous ribonucleoprotein particles (RNPs). RNPs consist of viral nucleoprotein, RNAs, and RNA-dependent RNA polymerase, and they associate with the non-structural protein NS4 to form inclusion bodies. These inclusion bodies interact with the microvilli of the host midgut epithelium, which are formed by actin, resulting in viral entry and propagation to midgut visceral muscles, alimentary tract, salivary glands, and reproductive system [65].

The rice yellow stunt virus (RYSV: *Rhabdoviridae*, negative-sense ssRNA), which is persistently transmitted by the leafhopper *N. cincticeps*, invades host nervous system and exploits axonal transport to disseminate throughout the leafhopper body. The neuron cytoskeleton structures consist of microtubules, actin filaments, and neuro-filaments. The interaction of RYSV with microtubule-based neurofilaments leads to the rapid delivery of virions along the axons, resulting in efficient viral dissemination [66].

#### 4.1.2. Horizontal Transmission of Plant Viruses through the Salivary Glands

Salivary glands are the ultimate transmission barrier for plant viruses before their transmission to a new host. RDV deficient in Pns10 failed to be transmitted to the plant secondary host by the vector *N. cincticeps* [67]. The RGDV tubules composed of Pns11 proteins interact directly with the actin-based apical plasmalemma of the salivary glands of *R. dorsalis* and release into the salivary cavities in an exocytosis-like process [68] (Figure 3). These results suggest that the interaction of viral non-structural proteins with the insect actin is primordial to breach through the salivary glands and be transmitted to a secondary host.

#### 4.1.3. Vertical Transmission of Plant Viruses

Vertical or transovarial transmission helps viral spread and persistence within vector populations. Vertical transmission requires that viral particles pass through the ovary follicular cells and enter the host oocyte [69]. The plasma membrane of follicular cells is connected with the oocyte through actin-based microvilli [70]. Some plant viruses use these actin-based junctions of the follicular cells to enter the oocyte. For instance, RGDV can enter oocytes of the *R. dorsalis* leafhopper through the actin-based microvilli of follicular cells as well as the actin-based junctions between the cells. Upon crossing the junctions between follicular cells, virions spread and propagate in the cytoplasm of the oocyte [71] (Figure 3).

Transovarial transmission of plant viruses can also be mediated by vitellogenin (Figure 3). Vitellogenin is synthesized in the fat body and transported into the growing oocytes through receptor-mediated endocytosis [72]. In the ovaries of the small brown planthopper, RSV expresses a nucleocapsid protein that binds to the vitellogenin. The vitellogenin-nucleocapsid complex moves along the follicular cells through actin-based junctions to enter the oocyte [72]. In addition, Pns10 and Pns11, which are non-structural proteins of RSV and RGDV, respectively, were found to interact with vitellogenin in the ovaries of the respective vectors. These interactions are involved in the vertical transmission of RSV and RGDV to the host progeny via receptor-mediated endocytosis into oocytes cytoplasm [69,71].

### 4.2. Arboviruses

Arboviruses represent an increasing threat to global veterinary and human health. Mosquitoes of the *Aedes* genus are the primary worldwide vectors of epidemic viruses such as Dengue (DENV: *Flaviviridae*, positive-sense ssRNA), Chikungunya (CHIKV: *Togaviridae*, positive-sense ssRNA), Zika (ZIKV: *Flaviviridae*) and yellow fever (YFV: *Flaviviridae*) viruses. Following a blood meal from an infected human, arboviruses replicate in the female midgut and further disseminate to reach the salivary glands. Once infection has been established in the salivary glands, it lasts throughout the mosquito lifespan, and arboviruses can be transmitted to subsequent hosts [73]. Seldomly, arboviruses can reach female ovaries and be vertically transmitted [74].

At present, most if not all our knowledge on arboviral interaction with the cytoskeleton of mosquito cells comes from descriptive transcriptomic and proteomic studies regarding the *Aedes albopictus* cell line C6/36 or *Aedes aegypti* females infected with either the alphavirus chikungunya virus (CHIKV; positive sense single-strand RNA virus) or flaviviruses (positive sense single-strand RNA viruses). These viruses share the fact that they enter host cells by a clathrin-dependent endocytosis pathway [75,76,77].

#### 4.2.1. Examples of Flavivirus Interaction with Insect Cytoskeleton

Dengue virus-labeled particles were detected within early endosomes 5 min post infection (mpi) and near lysosomes 0.5 hpi in C6/36 cells [75]. The disruption of host microtubule and microfilaments reduced viral infection by more than 80%, suggesting an implication of the cell cytoskeleton in the early steps of viral entry. In addition, 10 mpi with DENV, the co-localization of small groups of viral particles with host actin was detected, suggesting that actin filaments may favor movement of the viral particles [75]. At 48 hpi, transcription of actin and tubulin genes decreases to increase again at 96 hpi, suggesting a viral-mediated host shutoff effect followed by the activation of mechanisms that maintain homeostasis and reorganization of cytoskeleton during virus infection [78]. Further evidence on DENV manipulation of the host cell cytoskeleton comes from proteomic and transcriptomic analyses of different developmental stages of the mosquito *Ae. aegypti*. A total of 49 *Ae. aegypti* proteins were identified that interact with the DENV NS5 protein [79]. The highest enriched domains among these interacting proteins were found to be myosin-related [79]. In addition, several transcriptomic studies have shown that flavivirus infections alter the expression of genes in the cytoskeleton network such as actin, tubulin, and microtubule-associated proteins [77,80,81].

Similarly, west Nile virus (WNV) entry into C6/36 cells is drastically affected by chemical drugs that inhibit clathrin-mediated endocytosis or disrupt the microtubule network [77]. Additionally, the WNV non-structural protein NS2A and the envelope protein E were found to bind to cellular beta-tubulins, which build a network of microtubules [77]. However, the disruption of actin filaments did not affect the WNV entry into C6/36 cells [76].

#### 4.2.2. Examples of CHIKV Interaction with Insect Cytoskeleton

Proteomic analyses showed that the cytoskeleton-associated protein, syntenin, is induced as soon as 30 min after CHIKV infection in *Ae. aegypti* gut [82]. Syntenin is a membrane binding protein that regulates the architecture of the cell membrane, cellular trafficking, and is involved in actin polymerization [83,84]. In counterpart, actin and twinfilin, an actin-binding protein that regulates cytoskeleton dynamics, were repressed 30 mpi. However, the F-actin capping protein beta subunit, an actin-binding protein that regulates actin polymerization, was induced 24 hpi [82]. These results suggest a regulation of the actin polymerization dynamics at early time post CHIKV infection (0.5 to 24 hpi). Simultaneously with these observations, vesicle trafficking to the cellular vacuole was induced 0.5 hpi and was attenuated 24 hpi. Additionally, 24 hpi, endocytosis, vesicle formation and intracellular trafficking were reduced [82]. These results support the hypothesis that CHIKV manipulates the *Ae. aegypti* cytoskeleton by regulating actin polymerization/depolymerization. In addition to the interruption of endocytosis and cellular trafficking mechanisms after 24 hpi, tubulin and cytoskeleton motor proteins such as myosin were significantly downregulated in the mosquito gut [82]. This indicates a host response to restrain further de novo infection once the virus is spread throughout the mosquito tissues.

## 5. Conclusions

The currently available data on the manipulation of host cytoskeleton by viruses that infect insects suggest a primordial role of actin and microtubule cytoskeletons in viral internalization, replication, and dissemination. Common traits of cytoskeleton manipulation by insect viruses described in this review include the entry of viral particles through clathrin-mediated endocytosis, the direct interaction of viral proteins with actin, and possibly regulation of the expression of genes coding for components of the host cytoskeleton. Although the precise mechanisms of the interaction between insect viruses and host cytoskeleton are not well understood, current data suggest similar mechanisms as used by animal viruses. At least in the case of baculoviruses and plant reoviruses transmitted by insects, successful viral motility and dissemination depend on interactions of viral proteins with cytoskeleton proteins. Interestingly, amino acids and protein motifs intervening in the interaction with host cytoskeleton were identified, in primis, in the baculovirus protein family VP80 that shares common domains with host myosin-promotor proteins. Another common feature between these viruses is the formation during infection of tubular structures that interact with the actomyosin complex to transport the viral genetic material. In the case of arboviruses, several transcriptomic studies showed attenuation of the expression of cytoskeleton-related genes at different points post infection. Even though little is understood on whether and how insect viruses regulate the expression of host cytoskeleton-related genes, it is plausible to suggest that tight regulation of gene expression occurs simultaneously with the virus manipulation of actin and microtubule cytoskeletons.

A deeper understanding of the cellular and molecular mechanisms regulating cytoskeleton reorganization during viral infection of insect cells can have applicative outcomes. Depending on the insect–virus system and the application aims, cytoskeleton regulation might be enhanced or altered so that the viral infection is enhanced or blocked. In the case of insect pests, the knowledge of how infecting viruses hijack host cytoskeleton could be used to enhance viral pathogenesis toward more efficient biological pesticides that can compete with and replace chemical pesticides. A better understanding of the interaction between plant viruses and/or arboviruses and insect vector cytoskeleton may enable interfering with viral acquisition, retention, and transmission by the insect vectors, thus limiting or abrogating viral transmission to plants and/or animals/humans. As a result of the primordial role of cytoskeleton in cell function, it would be ideal to not directly target the host cytoskeleton but rather viral proteins that interact with or manipulate host cytoskeleton.
